# Therapeutical Potential and Immunomodulatory Profile of *Arthrospira platensis* Compounds against Chagas Disease

**DOI:** 10.1021/acsinfecdis.4c01052

**Published:** 2025-03-30

**Authors:** Maria Rafaele Oliveira Bezerra da Silva, Ana Carla da Silva, Byannca de Carvalho Torreão, Romero Marcos Pedrosa Brandão Costa, Raquel Pedrosa Bezerra, Silvana de Fátima Ferreira da Silva, Maria Luiza Vilela Oliva, Lícya Samara da Silva Xavier, Isabelle F.T. Viana, Roberto Dias Lins, Virginia Maria Barros de Lorena, Daniela de Araújo Viana Marques

**Affiliations:** † Integrated Multi-User Laboratory in Applied Biotechnology, Institute of Biological Sciences, University of Pernambuco, Santo Amaro, Recife, Pernambuco 50100-130, Brazil; ‡ Biotechnology and Parasitology Laboratory, University of Pernambuco, Santo Amaro, Recife, Pernambuco 50100-130, Brazil; § Department of Immunology, Aggeu Magalhães Institute, Fiocruz Pernambuco, Recife, Pernambuco 50670420, Brazil; ∥ Department of Animal Morphology and Physiology, Federal Rural of University of Pernambuco, Recife, Pernambuco 52171-900, Brazil; ⊥ Department of Biochemistry, Federal University of São Paulo (UNIFESP), São Paulo 04021-001, Brazil; # Departament of Virology, Aggeu Magalhães Institute, Fiocruz Pernambuco, Recife, Pernambuco 50670420, Brazil

**Keywords:** cyanobacteria, Trypanosoma cruzi, antiparasitic agents, cell survival, immunotherapy, cytokines

## Abstract

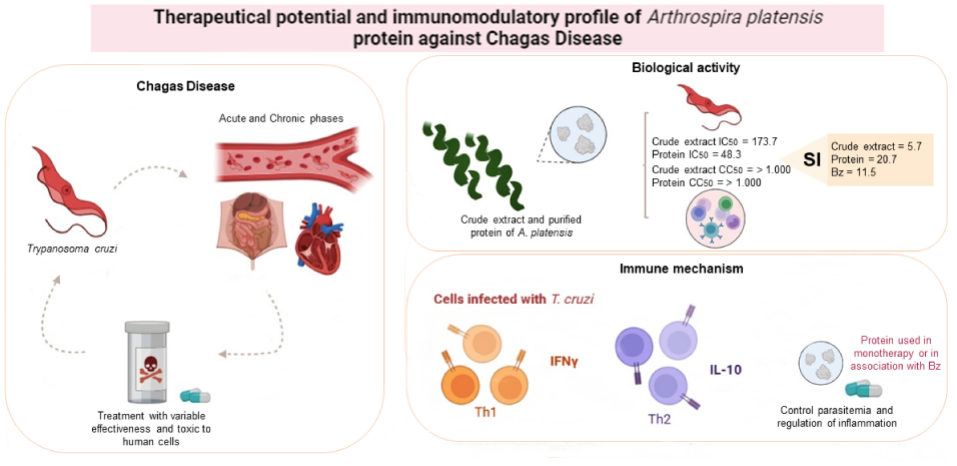

*Arthrospira platensis*, an ancient cyanobacterium, is rich in bioactive compounds with therapeutic potential, supporting its use in studies for various health conditions, including infectious and chronic diseases. This study aimed to evaluate the antiparasitic, cytotoxic, and immunomodulatory activities of *A. platensis* compounds against *Trypanosoma cruzi*. Peripheral Blood Mononuclear Cells (PBMC) and *T. cruzi* trypomastigotes were cultured for cytotoxic and antiparasitic analyses. Cytotoxicity was assessed in PBMC treated with different concentrations of crude extract, obtained by mechanical agitation in 0.1 M TRIS-HCl buffer (pH 7.2), and purified protein by DEAE-Sephadex A-50 chromatography and FPLC. Immune response was analyzed in infected and uninfected PBMC by measuring cytokines (IFN-γ, TNF, IL-2, IL-6, and IL-10) after treatment with purified protein and benznidazole. *In vitro* experiments showed that both crude extract and a 15 kDa purified protein were toxic to trypomastigotes in a dose-dependent manner, eliminating over 80% of the parasite at 1000 and 200 μg/mL, respectively. Both the extract and protein were nontoxic to PBMC, with the protein (SI: 20.7) being more selective than benznidazole (SI: 11.5). Results indicated that the purified protein modulated the immune response in *T. cruzi*-infected individuals, inducing a protective Th1 response while controlling an excessive inflammatory response with appropriate IL-10 levels. The anti-*T. cruzi* activity of this protein, alone or in combination with the commercial drug, suggests it could be a low-cost, safer, and more tolerable therapy for Chagas disease treatment.

Neglected diseases predominantly affect populations in developing regions, where health, housing, and food conditions are precarious. Research and development investments remain minimal, as these diseases offer limited financial returns and primarily impact low-income populations people.^1^ As a result, only 5.0% of the 1106 drugs introduced between 2000 and 2018 were developed for neglected diseases, emphasizing the critical need for novel therapeutic interventions.^2^

Among these neglected diseases, Chagas disease (CD), caused by the hemoflagellate *Trypanosoma cruzi*, is identified as a priority in the World Health Organization’s 2030 roadmap for neglected diseases.^3^ Although initially confined to Latin America, increased mobility and migration have transformed CD into a global public health concern.^4−6^ The disease often remains asymptomatic; however, in 30–40% of cases, patients progress to a chronic symptomatic phase with severe cardiac or digestive complications, often leading to mortality.^7−9^

Current pharmacological treatments for CD include nifurtimox and benznidazole (Bz), which exhibit variable efficacy based on patient demographics, disease stage, dosage, and treatment duration, with greater effectiveness observed in the acute phase.^10^ These drugs are further limited by significant cytotoxicity and the emergence of resistant *T. cruzi* strains.^11,12^ The absence of safe and effective therapeutic options underscores the urgent need to expand the arsenal against *T. cruzi*, focusing on compounds with low toxicity and minimal resistance induction.

Natural products, particularly those derived from photosynthetic organisms as *Arthrospira platensis*, a cyanobacterium recognized as GRAS by the FDA,^13^ are promising candidates for drug development.^14^ This cyanobacterium, utilized as a food source by indigenous communities around Lake Chad in Africa and in regions of Mexico and Central America since ancient times, is rich in bioactive compounds, including proteins, alkaloids, vitamins, peptides, and pigments, with documented medicinal properties.^15,16^ While its antiparasitic potential remains underexplored, its anti-inflammatory and immunomodulatory activities suggest potential efficacy against *T. cruzi*.^17^

Recently, Abdellatief et al.^18^ demonstrated that a crude extract of *A. platensis* modulates the immune system *in vivo* by stimulating Th1 cytokines and nitric oxide synthesis. Similarly, *A. maxima*, a related species within the Oscillatoriales order, was shown to enhance proinflammatory cytokine synthesis and ROS production, reducing parasite loads in *T. cruzi*-infected mice.^19^ These pathways are crucial for *T. cruzi* elimination, as they impair parasite proliferation by inducing DNA damage.^20^ Furthermore, modulating the immune response and enhancing host resistance may support clinical improvement in affected patients.^21^

While no studies have directly reported trypanicide activity for *A. platensis*, its antiparasitic effects against other trypanosomatids are documented. An aqueous extract of *A. platensis* inhibited *Leishmania infantum* promastigotes *in vitro* with a selectivity index of 3.8.^22^ However, the composition of the crude extract and the bioactive compounds responsible remain unidentified, underscoring the need for isolation and purification to explore its therapeutic potential. Therefore, we investigated the antiparasitic effects and immunomodulatory potential of *A. platensis* bioactive compounds, aiming to elucidate their mechanisms of action and explore their potential as an innovative approach for the treatment of Chagas disease, with the prospect of overcoming the limitations of conventional therapies.

## Results and Discussion

### *A. platensis* Protein Purification and Characterization

**Figure 1:**
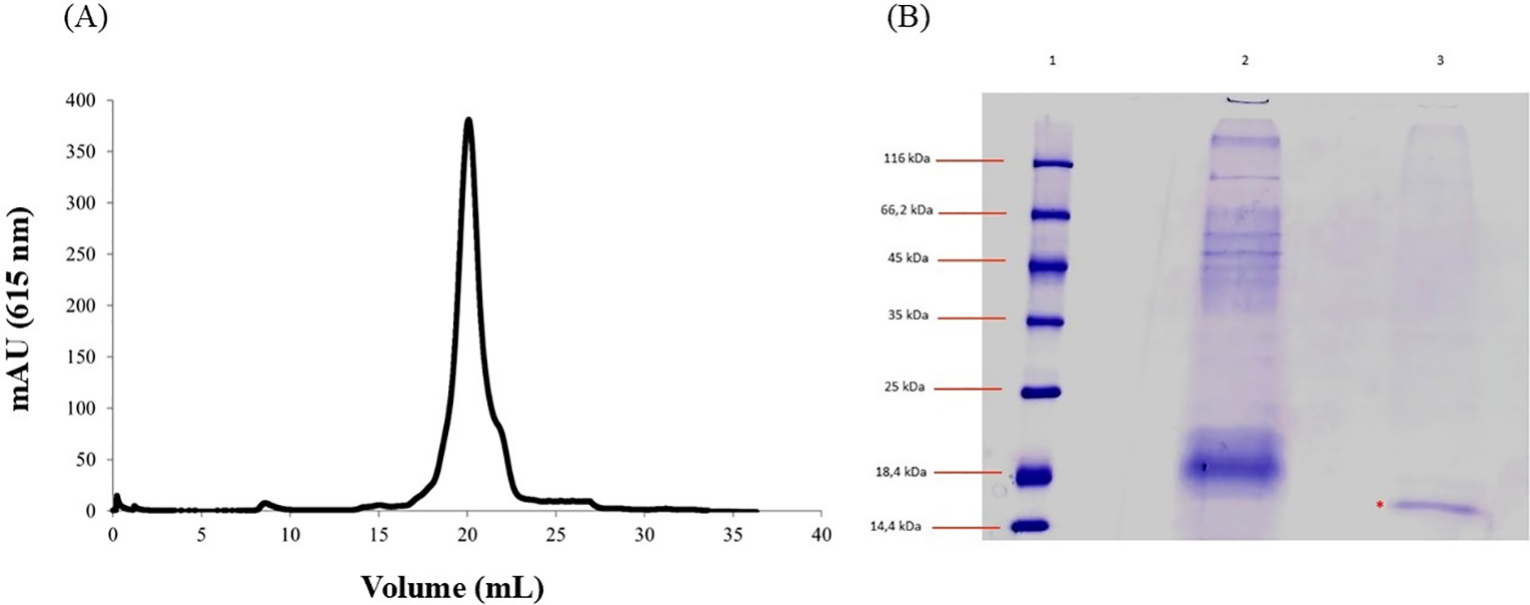
Protein purification. (A) Elution profile of the protein of the crude extract from *A. platensis*, obtained using FPLC by size-exclusion column monitored at 615 nm. (B) SDS–PAGE profile of protein. Lanes 1: SigmaMarker, 14,4 to 116 kDa mol. wt. ladder. Lanes 2: Crude extract. Lane 3: *A. platensis* nonadsorbed fraction recovered by anion exchange chromatography.

The crude extract of *A. platensis*, at a concentration of 568 μg/mL, was loaded onto an ion exchange column for a purification step. The nonadsorbed fraction that was collected showed a total protein concentration of 251 μg/mL. A sample of this fraction was injected onto a molecular exclusion column, and the resulting chromatogram displayed a single peak (Figure 1A), indicating the presence of a purified protein, whose molecular mass, determined by SDS-PAGE analysis, is 15 kDa (Figure 1B).

In *A. platensis*, these 15 kDa bands could correspond to small subunit of RuBisCO (ribulose-1,5-bisphosphate carboxylase/oxygenase) a photosynthetic enzyme that plays the main role in CO_2_ biofixation. RuBisCO itself is not widely recognized for direct health benefits, and there is a lack of studies investigating its therapeutic potential in humans.^23−25^ To the best of our knowledge, this represents the first study to investigate the antiparasitic potential of this protein.

### PBMC Viability in the Presence of *A. platensis* Crude Extract and Purified Protein

**Figure 2:**
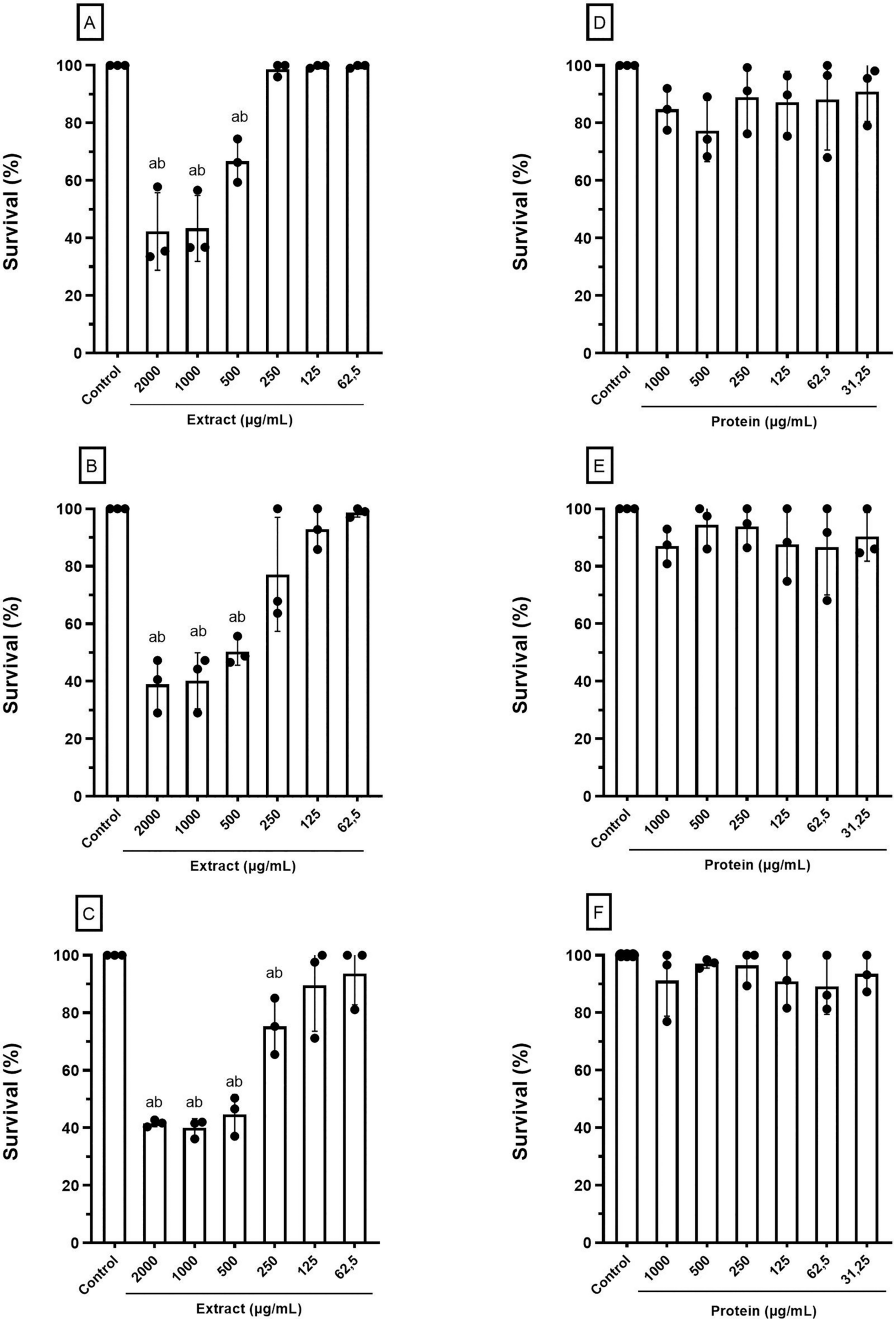
Effects of *A. platensis* crude extract (A–C) and 15 kDa purified protein (D–F) on the viability of human peripheral blood mononuclear cells (PBMC) after exposure for 24 (A and D), 48 (B and E), and 72 h (C and F). Negative control (untreated cells). Different letters (ab) indicate significant differences with the negative control by ANOVA (*p* ≤ 0.05).

Cytotoxicity assays were conducted to compare the potential harmful effects of the *A. platensis* crude extract and the 15 kDa purified protein on PBMC at 24, 48, and 72 h. At the highest concentrations, the crude extract reduced cell viability by 50% (*p* ≤ 0.05), but no significant cytotoxicity was observed at lower concentrations, particularly at 24 h (Figure 2A–C). The purified protein exhibited no major toxic effects, with cell survival rates comparable to controls (Figure 2D–F).

Although *A. platensis* is classified as a GRAS product, safety data on its extracts and proteins remain limited. Vaitkevicius-Antão et al.^22^ reported a CC_50_ of 986.1 μg/mL for an *A. platensis* aqueous extract in PBMC—aligning with our findings (CC_50_ > 1000 μg/mL). Notably, PBMC—showed greater tolerance to the purified protein, with a maximum safe dose of 3.0 mg/mL (CC_50_ > 1000 μg/mL). Previous studies on *A. platensis* peptides demonstrated no toxicity to healthy cells within a wide concentration range after 24 h, but longer exposure effects were not assessed.^26,27^ Our findings confirmed that both the crude extract and the 15 kDa protein were nontoxic—in all tested time points, highlighting their potential as safe therapeutic agents for human applications.

### Effects of *A. platensis* Crude Extract and 15 kDa Protein on *T. cruzi* Viability

**Figure 3:**
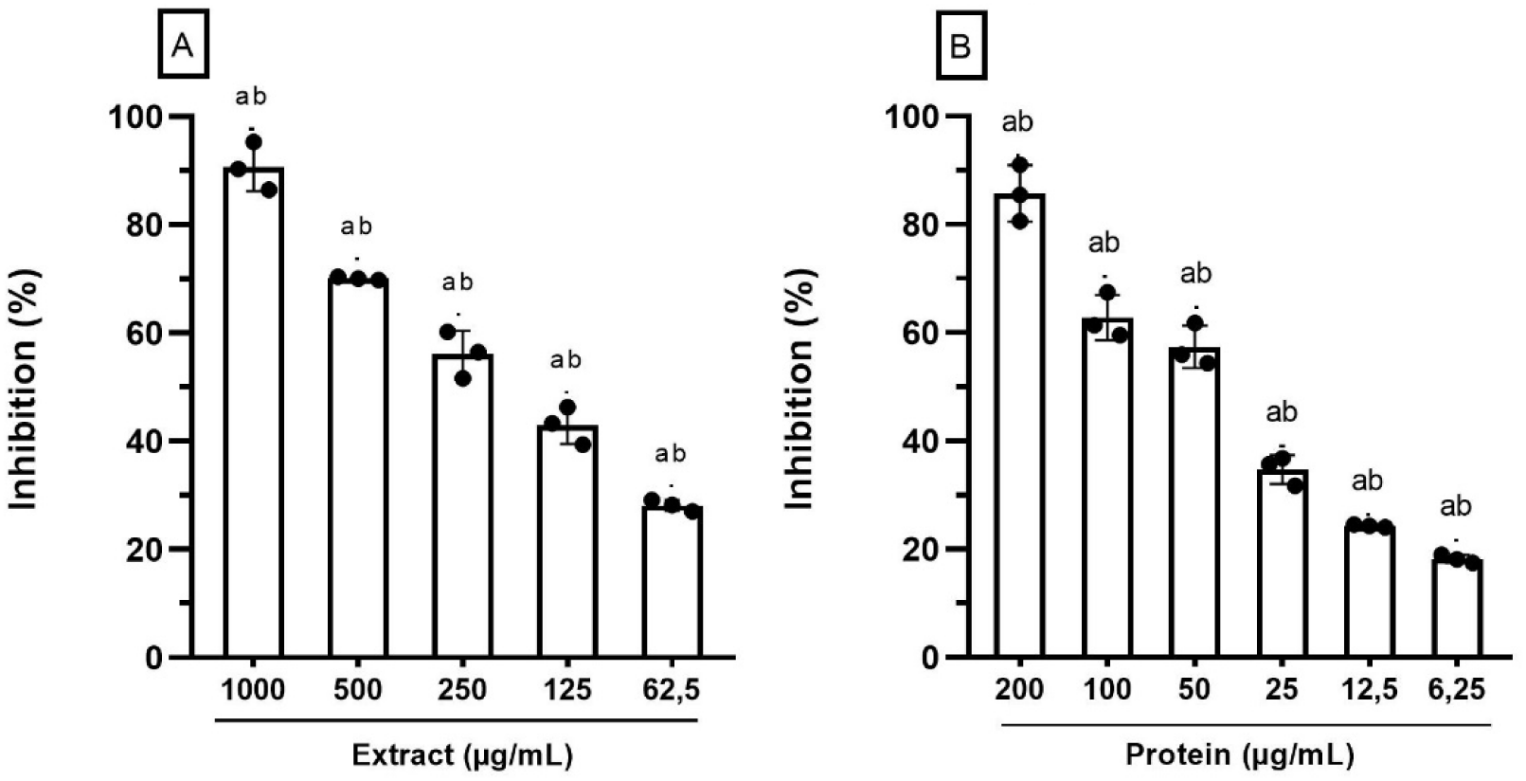
Effects of the crude extract (A) and protein (B) from *A. platensis* on *T. cruzi* trypomastigote viability. Different letters (A,B) indicate significant differences between treatments by ANOVA (*p* ≤ 0.05).

The crude extract and the isolated protein showed dose-dependent antiparasitic effects on trypomastigote forms. The crude extract (1000 μg/mL) and the isolated protein (200 μg/mL) showed significant toxic activity against trypomastigotes, affecting parasite viability by more than 80.0% (*p* ≤ 0.0001). At lower concentrations, starting from 62.5 μg/mL, the crude extract of *A. platensis* reduces activity against *T. cruzi*. However, even at low concentrations, the isolated protein showed trypanicide activity (Figure 3).

Most studies investigating drugs for Chagas disease use natural compounds of plant origin. However, little trypanicide activity investigations have been reported among photosynthetic microorganisms. Recently, only one study reported the action of *A. platensis*, but the organic and aqueous extracts used did not inhibit parasite viability, when tested at a concentration of 200 μg/mL.^28^ Furthermore, the aqueous extract has also been used to analyze the antiparasitic activity against other trypanosomatids, such as *Leishmania infantum*, demonstrating leishmanicide activity when tested at concentrations between 15.6 to 500 μg/mL.^22^ Our findings corroborate the antiparasitic activity of the crude extract and isolated protein highlighted by Vaitkevicius-Antão et al.,^22^ demonstrating the effect on *T. cruzi*, with IC_50_ of 173.7 and 48.3 μg/mL, respectively.

Studies with crude extract or proteins isolated from *A. platensis* are scarce. Our data indicate that the activity of the purified protein is higher than that reported by other studies that evaluated natural compounds.^29^ Likewise, the purified protein from *A. platensis* showed greater activity than the *Chlorella vulgaris* extract, which presented moderate toxicity for *T. cruzi* (IC_50_ = 112.10 μg/mL).^30^ A peptide isolated from the cyanobacterium *Oscillatoria nigroviridis* was effective against *T. cruzi*, with an IC_50_ of 1.1 μM.^31^ However, unlike the present study, the cytotoxicity of *O. nigroviridis* peptide against healthy cells has not been reported, making it difficult to consider this compound for the future development of drugs for the treatment of CD (because it is still not possible to guarantee the safety of treated patients).

### Selectivity Index of the *A. platensis*Purified Protein

The relationship between cytotoxicity and the anti-*T. cruzi* activity was determined by calculating the Selectivity Index (SI) (Cytotoxicity CC_50_/*T. cruzi*IC_50_). As a standard, high SI values indicate that a compound is more toxic to a parasite than to host cells, and SI values below 10.0 indicate that the tested compound is toxic to healthy cells.^32^ Recently, Veas et al.^28^ analyzed the activity of organic extracts from three microalgal species (*Chlamydomonas reinhardtii*, *Tetraselmis suecica*, and *Scenedesmus obliquus*). All these microalgae exhibited high anti-*T. cruzi* inhibition rates, but were considered toxic to Vero cells due to their significantly low selectivity indexes: *C. reinhardtii*, SI = 3.3; *T. suecica*, SI = 4.8; and *S. obliquus*, SI = 2.9. The extracts of *C. vulgaris* and *T. obliquus* were more selective for *T. cruzi* trypomastigotes, showing SI of 8.9 and 16.8, respectively (Silva-Júnior et al., 2024).

The crude extract of *A. platensis* analyzed in this study was shown to be slightly more selective than these microalgae *C. reinhardtii*, *T. suecica*, and *S. obliquus* after 24 h of treatment (SI = 5.7). However, after protein purification, the SI significantly improved, reaching a value of 20.7, therefore being superior to all supported treatments. Bz showed an SI of 11.5, as evaluated by treating healthy cells at concentrations ranging from 0.5 to 8.0 μg/mL and trypomastigotes at concentrations ranging from 0.25 to 4.0 μg/mL (data not shown). Therefore, the purified protein showed the best SI among the evaluated compounds, including the commercial reference drug, suggesting that this protein could be an interesting alternative for new drugs in treating CD.

### PBMC Immune Responses in the Presence of the*A. platensis*Purified Protein

**Figure 4:**
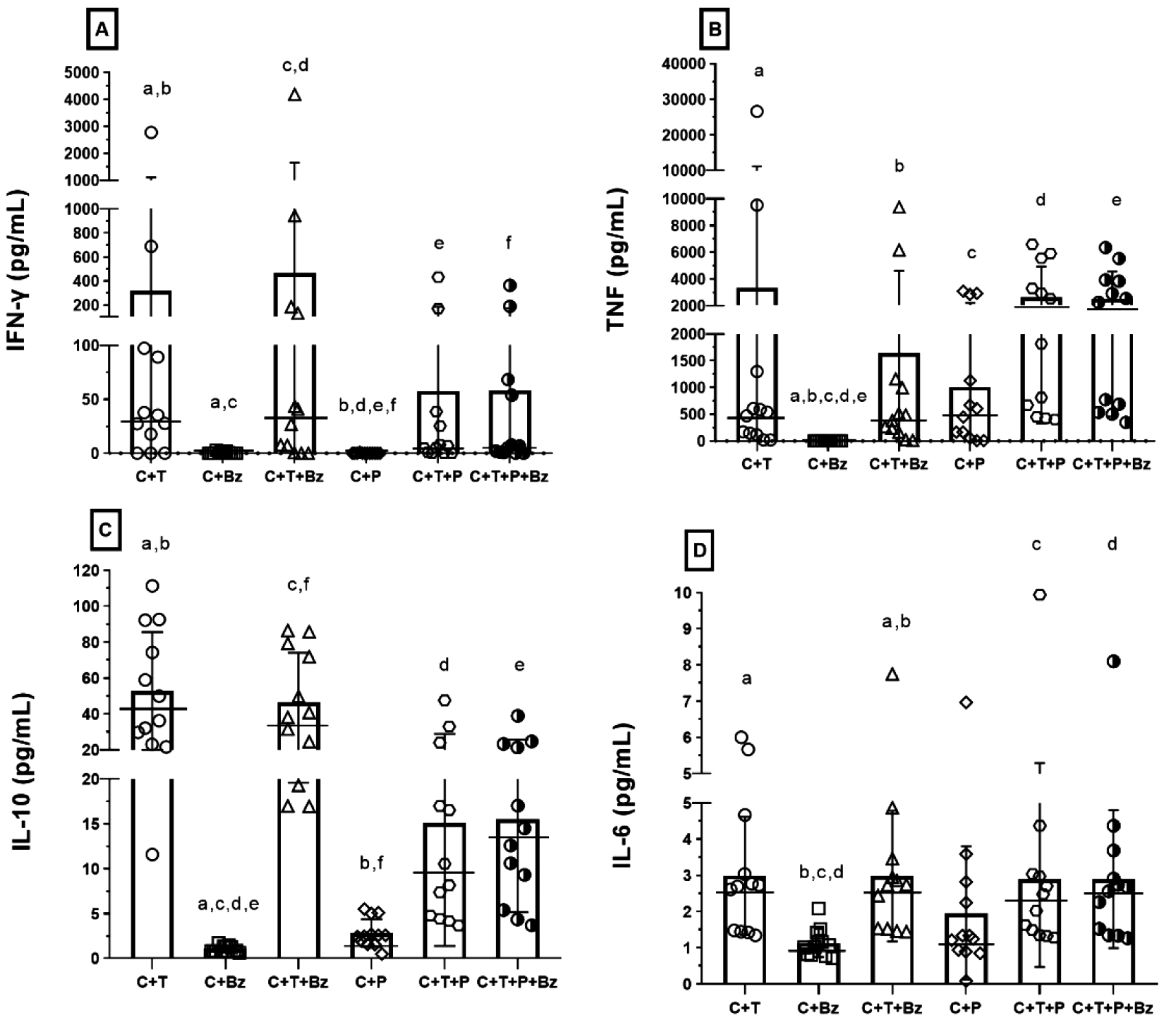
Effect of the*A. platensis*purified protein at a concentration of 48.3 μg/mL on the production of different cytokines by PBMC infected or not with *T. cruzi* trypomastigotes after 24 h exposure (A-D). The treatments were: uninfected PBMC—treated with Bz at 1.0 μg/mL (C + Bz) and infected PBMC treated with Bz at the same concentration (C + T + Bz); Uninfected PBMC—treated with protein at 48.3 μg/mL (C + P) and infected PBMC treated with protein at the same concentration (C + T + P); Infected PBMC—treated with protein and Bz (C + T + P + Bz). Equal letters between treatments represent significant differences between them by ANOVA or Kruskal–Wallis (*p* ≤ 0.05).

Previous reports have shown that compounds isolated from algae can act as immunomodulatory agents. This is interesting from a therapeutic point of view since, by modulating the production of cytokines and other immune mediators, these bioactive products can enhance the defense system against infectious diseases.^33^ In this study, we evaluated the effect of the purified protein isolated from *A. platensis* (48.3 μg/mL) and Bz (1.0) on cytokine release by infected and uninfected PBMCs (Figure 4).

The protein induced the production of pro-inflammatory cytokines (IFN-γ, TNF, and IL-6) in noninfected cells (at levels higher than those observed in cells treated only with Bz) and exhibited a modulatory effect in *T. cruzi*-infected cells. In these cells, which displayed elevated cytokine levels due to the host’s natural inflammatory response to the infection, the protein, both alone and in combination with Bz, significantly attenuated expression of these cytokines. It is well-established that, particularly IFN-γ and TNF, are cytotoxic mediators associated with the Th1 immune response, whose activation is critical for controlling the viability of the microorganism. Clinical studies have shown that individuals in the acute phase of the disease have a robust inflammatory response, producing inflammatory cytokines, such as IFN-γ and TNF, that activate macrophages to eliminate the parasite.^34,35^ However, excessive stimulation of these pro-inflammatory cytokines could harm the host by causing exacerbated inflammatory responses.^36^ Therefore, the effect induced by the protein suggests that, although it stimulates cytokine production in noninfected cells, it plays a regulatory role under conditions of exacerbated inflammation, contributing to the attenuation of the inflammatory response in *T. cruzi*-infected cells. At a concentration of 48.3 μg/mL, the protein did not stimulate the release of IL-2 in infected cells (data not shown).

In contrast, the protein stimulated IL-10 secretion, an anti-inflammatory cytokine produced by T cells and monocytes that inhibits pro-inflammatory cytokines.^37^ In noninfected cells, the protein induced higher IL-10 levels than Bz, indicating its role in promoting a Th2 response (Figure 4C). However, in infected cells, IL-10 expression was lower than in untreated or Bz-treated infected cells. However, these findings suggest a potential advantage of this new protein, since high IL-10 expression facilitates *T. cruzi* evasion by inhibiting macrophage activation induced by IFN-γ.^38^ These results suggest that the combination treatment in infected cells was well tolerated without impairing immunomodulation. This highlights the therapeutic potential of both the protein alone and in combination for Chagas disease treatment. Control by means of immunoregulatory mechanisms is extremely important to prevent the deleterious effects associated with the excessive inflammatory response, which are directly linked to the main consequences of the morbidity characteristic of Chagas disease, such as the progression of heart disease.^39,40^

These findings were consistent with other studies suggesting that*A. platensis*may have beneficial effects in modulating the immune response. Its extract has been indicated in the literature for leading to reduced production of pro-inflammatory cytones.^41−43^ Regarding the production of IL-10, our results agree with those obtained by Vaitkevicius-Antão et al.^22^ that when treating human PBMC—with the aqueous extract of *A. platensis*, showed that the cytokine was considerably stimulated at a concentration 4x lower than the CC_50_ determined in the study, as demonstrated in our study when healthy cells were treated with 48.3 μg/mL. In contrast, in infected cells, a reduction in the expression of this cytokine was observed, similarly to what was shown in the study by Mahmoud et al.^44^ in which the release of IL-10 was attenuated after the cells were infected with a virulent strain of *Pseudomonas fluorescens*.

In addition, we classified individuals as ″high″ or ″low″ cytokine producers based on a cutoff derived from the global average cytokine production.^45^ The frequency of high cytokine producers was evaluated for chagasic individuals following *in vitro* stimulation with protein, benznidazole, and their combination. Table 1 shows the frequency of high cytokine producers across treatments.

**Table 1:** Frequency of Cytokine High-Producer Subjects Based on the Global Median Cytokine Cut-Off Detected After Stimulation with Treatments

		C+T	C + Bz	C + T + Bz	C + P	C + T + P	C + T + P + Bz
**IFN-γ**	**Global median cutoff**	31.6 (0.0–2.783)	0.0 (0.0–2.6)	34.0 (0.0 – 4.194)	0.0 (0.0–0.9)	6.1 (0.0–432.3)	6.0 (0.0–362.7)
	**High cytokine producers (%)**	41.6%	0.0%	41.6%	0.0%	50.0%	50.0%
**TNF**	**Global median cutoff**	509.5 (23.0–26.659)	1.3 (0.0–8.0)	439.4 (13.26–9.384)	530.4 (7.4–3.101)	2.177 (404.2–6.594)	2.407 (347.5–6.355)
	**High cytokine producers (%)**	50.0%	58.3%	50.0%	50.0%	50.0%	58.3%
**IL-10**	**Global median cutoff**	43.1 (11.5–111.4)	0.9 (0.6400–1.7)	39.4 (16.9–86.5)	2.5 (0.5–5.5)	9.3 (3.6–47.7)	13.5 (3.6–39.0)
	**High cytokine producers (%)**	50.0%	0.0%	58.3%	66.6%	50.0%	50.0%
**IL-6**	**Global median cutoff**	2.7 (1.3–6.0)	1.0 (0.7–2.0)	2.7 (1.4–7.7)	1.2 (0.09–6.9)	2.2 (1.2–9.9)	2.6 (1.2–8.1)
	**High cytokine producers (%)**	58.3%	58.3%	58.3%	66.6%	50.0%	50.0%

Data are expressed as the percentage of individuals displaying cytokine+ cells higher than or equal to the global median (cutoff) calculated for each cell population. PBMC infected with *T. cruzi* (C + T), with Bz (C + Bz) or protein (C + P), infected and treated with Bz (C + T + Bz) or protein (C + T + P), and infected and treated with Bz and protein in association (C + T + P + Bz). The chi-square test was used, and statistical significance (*p* ≤ 0.05) is represented by the superscript symbol * for comparisons between treatments.

The results showed that after treatment with protein and Bz, 58.3% of individuals were high TNF producers. For IL-6, 66.6% were high producers, predominantly after protein treatment. Regarding IL-10, 66.6% of individuals treated with protein were high producers. No high producers of IFN-γ were found across any treatments, but 50.0% of individuals were high producers when treated with protein alone or in combination.

Finally, we analyzed the correlation of the expression of these cytokines between the treatments and observed that for the secretion of IFN-γ, there was a very strong and significant positive correlation (*r*= 0.931; *p* ≤ 0.0001) between the treatments with the protein in monotherapy and in association with Bz in infected cells. These treatments also showed a strong and significant correlation for TNF, IL-10, and IL-6 secretion. In the same manner, treatment with the drug alone also showed a positive and significant correlation compared to treatment in association with the protein (*r*= 0.979; *p* ≤ 0.0001). In contrast, for TNF stimulation, treatment with the protein in healthy cells showed a very strong negative correlation with treatment with Bz in infected cells (*r*= −0.301). However, there were no significant differences (*p* = 0.342). The other treatments showed moderate or very strong positive correlations, but no significant differences. These data can be seen in Table 2.

**Table 2:** Coefficient of Correlation Enters the Cytokines Produced After the Stimulus with the Different Treatments in 24 h of Treatment^a^

	C + T + Bz x C + P	C + T + Bz x C + T + P	C + T + Bz x C + T + P + Bz	C + P x C + T + P	C + P x C + T + P + Bz	C + T + P x C + T + P + Bz
	**IFN-γ**					
*r*	0.306	0.938	0.979^*^	0.308	0.219^*^	0.931^*^
*p*-value	0.500	0.266	*p* ≤ 0.0001	0.500	*p* ≤ 0.0001	*p* ≤ 0.0001
	**TNF**					
*r*	–0.301	0.402	0.573	0.427	0.280	0.944^*^
*p*-value	0.342	0.177	0.056	0.169	0.379	*p* ≤ 0.0001
	**IL-10**					
*r*	0.256	0.510	0.699^*^	0.547	0.453	0.769^*^
*p*-value	0.418	0.094	0.014	0.069	0.141	0.005
	**IL-6**					
*r*	0.606^*^	0.839^*^	0.867^*^	0.750	0.743	0.965^*^
*p*-value	0.040	0.001	0.001	0.007	0.007	*p* ≤ 0.0001

a Pearson or Spearman correlation test was utilized to evaluate correlations among cytokines, and the ^*^ represents statistical differences with the value of p ≤ 0.05. PBMC infected with *T. cruzi* (C + T), with Bz (C + Bz) or protein (C + P), infected and treated with Bz (C + T + Bz) or protein (C + T + P), and infected and treated with Bz and protein in association (C + T + P + Bz).

Correlation tests are important for analyzing the association between two cytokines. These results suggest that both treatments with positive and significant correlations, in particular, may have similar or highly correlated effects on infection control, so that both treatments may be acting on similar pathways in the cells. Therefore, both, the protein administered as monotherapy and its association with Bz, could potentially confer therapeutic benefits for individuals affected by Chagas disease, since they generated an immune response similar to the standard drug, which is effective against *T. cruzi*, but even more robust than this drug, which could allow the necessary dose of each individual treatment to be reduced, thus minimizing the associated side effects and promoting a safer and more tolerable therapy for patients.

## Conclusion

In the present study, crude extract and purified protein from *A. platensis* showed anti-*T. cruzi* activity against the trypomastigote form of the parasite. Notably, at low concentrations, the 15 kDa protein inhibited the viability of these evolutionary forms by more than 80%, without leading to the death of healthy human cells, with a safe dose of 3.0 mg/mL. In addition, our results indicated that treatment with the protein can modulate the immune response of individuals infected with *T. cruzi*, inducing a protective Th1 response, while also stimulating IL-10 at appropriate levels to control an exacerbated inflammatory response, which could cause damage to the patient’s tissues. *In vitro* analyses indicate that, the anti-*T. cruzi* activity of this protein in monotherapy or in association with Bz suggests that the compound may be able to control parasitemia while regulating inflammation and preventing the progression of heart disease. These findings provide promising insights for the development of more effective and targeted therapeutic strategies in the context of Chagas disease treatment.

## Experimental Section

### Microorganisms and Culture Conditions

*A. platensis* UTEX, 1926 was obtained from the University of Texas Culture Collection (Austin, TX, USA) and cultivated in SAG medium^46^ at an initial concentration of 50 mg/mL. The culture was maintained on an orbital shaker under autotrophic conditions, and exposed to continuous light (72 ± 5 μmol photons/m^–2^/s) until the exponential growth phase was reached (after 7 days).

### Preparation of the Extract

The extract was prepared according to the methodology described by Gago et al.,^47^ with some modifications. Briefly, the biomass was collected by centrifugation at 4500 rpm at 4 °C for 10 min. The pellet was then washed in ddH_2_O and centrifuged under the same conditions, frozen at −80 °C, and lyophilized to obtain dried samples. The extract was obtained by dissolving 1 mg of the biomass in Tris-HCl 0.1 M (pH 7.2), stirring for 9 h, and centrifuging at 4500 rpm at 4 °C for 5 min. The supernatant (crude extract) was recovered and stored at −20 °C until use.

### Protein Purification and Characterization

#### Protein Purification

An aliquot (1.0 mg/mL) of the crude extract was subjected to chromatography using DEAE-Sephadex A-50 (a weak ion changer anionic resin) (Cytiva, USA) packed in a glass column (20 × 300 mm). The column was equilibrated and washed with 500 mL of 0.1 M biphasic phosphate buffer (pH 7.5). The elution procedure for unbound proteins was performed at a flow rate of 1.0 mL/min using 0.1 M biphasic phosphate buffer (pH 7.5) without salt addition; fractions (1 mL) were collected and monitored at 280 nm using a spectrophotometer. Subsequently, size-exclusion analysis of the purified proteins was performed using an AKTA purifier 10 by fast performance liquid chromatography (FPLC) system (Cytiva, USA) equipped with a GE Superdex 75 10/300 GL column. The run was monitored at 615 nm, and 1 mL fractions were collected using an automatic fraction collector. The peaks were then subjected to detection of cytotoxic and antiparasitic activities.

#### Protein Characterization and Concentration Estimation

Purified proteins were characterized by 15% SDS-PAGE under nondenaturing conditions,^48^ and their sizes were determined using molecular weight markers (14.4 – 116 kDa). Protein concentration was assessed using the BCA Protein Assay Kit, with a calibration curve constructed using serum albumin (0 to 2000 μg/mL) as standard.

### Parasite and Cell Viability Analysis

#### Obtaining Human Peripheral Blood Mononuclear Cells (PBMC)

Blood samples were collected from three individuals in sodium heparin tubes (The proposal was approved by the Human Research Ethics Committee – CAAE: 30184720.6.0000.5191). Under sterile conditions, blood was diluted 1:1 with phosphate-buffered saline (PBS, pH 7.2). To isolate PBMCs, the blood-PBS mixture was layered over Ficoll-Hypaque solution (1:1 ratio) and centrifuged at 900 × g for 30 min at 22 °C without brake. The PBMC layer was collected and washed twice in sterile PBS (pH 7.2) by centrifugation at 400 × g for 10 min at 22 °C. The pellet was resuspended in 1 mL of RPMI 1640 medium supplemented with 10% fetal bovine serum (FBS) and 100 μg/mL streptomycin. The cell concentration was adjusted to 2 × 10^6^ cells/mL using RPMI 1640 medium with 10% FBS for subsequent use.

#### Cultivation of the Trypomastigote Form of *T. cruzi*

*T. cruzi* trypomastigotes (Y strain) were cultured in Vero cells at 37 °C with 5% CO_2_ in RPMI 1640 medium containing 10% FBS and streptomycin. After approximately 6 days, cell rupture released the trypomastigotes, which were collected by centrifugation (3000 rpm, 10 min). The pellet was resuspended in RPMI 1640 medium with 10% FBS, and parasites were counted and adjusted to 1 × 10^6^ trypomastigotes/mL for further antiparasitic activity analysis.

#### Cytotoxic Assay in Healthy Cells

PBMC from healthy humans were incubated in 96-well microplates and treated with different stimuli to evaluate the 50% cytotoxic concentration (CC_50_) in human cells according to Mosmman,^49^ with some modifications. Each treatment (containing 2 × 10^6^ cells/mL) was performed in triplicate. Cells were incubated for 24, 48, and 72 h in the presence of 62.5 to 2000 μg/mL of *A. platensis* crude extract and 31.2 to 1000 μg/mL of the protein purified in this study. Untreated cell samples, in triplicate, were used as negative controls. At the end of each culture time, the supernatant was discarded, MTT [3-(4,5-dimethylthiazol-2-yl)-2,5-diphenyltetrazolium bromide] was added at a concentration of 5 mg/mL, and samples were incubated in an oven at 37 °C for 3 h. Subsequently, the formazan crystals formed were solubilized in dimethyl sulfoxide (DMSO), and the optical density of the solution was determined with a spectrophotometer at 540 nm.

#### Cytotoxic Assay in Trypomastigote Form

The trypomastigote forms Y strain (10^6^ parasites/mL) obtained from the supernatant of VERO cells were treated with the crude extract of *A. platensis* at concentrations of 31.2 to 1000 μg/mL and 6.2 to 200 μg/mL of the purified protein. After 24 h of treatment, the number of viable parasites with apparent motility was determined by direct counting in a Neubauer chamber to determine the IC_50_ (Concentration capable of reducing the number of trypomastigotes by 50%). The results were quantitatively verified using the method described by Rashed et al.^50^

#### Immune Response Analysis

To analyze the immune response stimulated by the protein, whole blood was collected from 12 healthy patients to obtain PBMC. These cells were cultured in 96-well microplates (2 × 10^6^ cells/well), which were incubated at 37 °C for 4 h to allow adherence of the cells (mainly monocytes). Next, trypomastigotes (2 × 10^5^ cells) were added to the corresponding wells. The plates were incubated (37 °C, 5% CO_2_) for 24 h to allow the infection of PBMC. Subsequently, all wells were treated with 15 kDa protein at 48.3 μg/mL or Bz (1.0 μg/mL) or protein + Bz. The plates were incubated (37 °C, 5% CO_2_) for 24 h, and the supernatant from each well was removed and immediately stored at – 80 °C for later use to measure cytokine levels. The ratio between the cytokines produced by cells under treatment and those produced by nonstimulated cells was calculated.

#### Cytokine Production Assay

Cytokine secretion was measured in human PBMC culture supernatants. The assay was performed using a BD CBA Human Th1/Th2 Cytokine Kit (Becton Dickinson, USA), and interferon (IFN) γ, tumor necrosis factor (TNF), interleukin (IL) 2, IL-6, and IL-10 cytokines were measured. Readings were performed using a FACSCalibur flow cytometer (Becton Dickinson, USA) according to the manufacturer’s guidelines. The results were analyzed using FCAP Array software (v3.0; Becton Dickinson, USA) and normalized to the results obtained using nonstimulated cells.

#### Data Analysis

The data were analyzed using descriptive statistics, including absolute and percentage distributions. CC_50_ and IC_50_ values were determined via nonlinear regression analysis (log inhibitor vs normalized response). The selectivity index (SI), reflecting parasite toxicity relative to cytotoxicity, was calculated as SI = log [CC_50_]/[IC_50_]. The Shapiro-Wilk test assessed normality, followed by one-way ANOVA with Tukey’s posthoc test (parametric) or Kruskal–Wallis with Dunn’s posthoc test (nonparametric) for group comparisons. Chi-square tests evaluated cytokine production categories, and Pearson or Spearman correlation tests assessed cytokine correlations. All analyses were conducted using GraphPad Prism 8.0.1, with *p* ≤ 0.05 considered significant.

## References

[ref1] Torreele E.; Usdin M.; Chirac P. A Needs-Based Pharmaceutical R&D Agenda For Neglected Diseases; 2004, Vol: 31. www.dndi.org. accessed 15 December 2024.

[ref2] Ferreira L. L. G.; Andricopulo A. D. Drugs and vaccines in the 21st century for Neglected Diseases. Lancet Infect. Dis. 2019, 19 (2), 125–127. 10.1016/S1473-3099(19)30005-2. 30712832

[ref3] World Health Organization. Accelerating work to overcome the global impact of Neglected Tropical Diseases a roadmap for implementation, 2012. https://apps.who.int/iris/bitstream/handle/10665/338712/WHO-HTM-NTD-2012.5-ebg.pdf. accessed 15 December 2024.

[ref4] Bern C.; Montgomery S. P. An estimate of the burden of Chagas Disease in the United States. Clin. Infect. Dis. 2009, 49 (5), e52–e54 10.1086/605091. 19640226

[ref5] Lee B. Y.; Bacon K. M.; Bottazzi M. E.; Hotez P. J. Global economic burden of Chagas Disease: A computational simulation model. Lancet Infect. Dis. 2013, 13 (4), 342–348. 10.1016/S1473-3099(13)70002-1. 23395248 PMC3763184

[ref6] World Health Organization. Control of Chagas Disease. 2002. World Health Organization technical report series 905, 1–109, https://apps.who.int/iris/handle/10665/42443. accessed 15 December 2024. 12092045

[ref7] Teixeira A. R. L.; Nitz N.; Guimaro M. C.; Gomes C.; Santos-Buch C. A. Chagas Disease. Postgrad. Med. J. 2006, 82 (974), 788–798. 10.1136/pgmj.2006.047357. 17148699 PMC2653922

[ref8] Castillo-Riquelme M. Chagas Disease in non-endemic countries. Lancet Glob Health 2017, 5 (4), e379–e380 10.1016/S2214-109X(17)30090-6. 28256341

[ref9] Pérez-Molina J. A.; Molina I. Chagas Disease. Lancet 2018, 391 (10115), 82–94. 10.1016/S0140-6736(17)31612-4. 28673423

[ref10] Coura J. R.; Castro S. L. D. A critical review on Chagas Disease chemotherapy. Mem. Inst. Oswaldo Cruz. 2002, 97 (1), 3–24. 10.1590/S0074-02762002000100001. 11992141

[ref11] Andrade H. M.; Murta S. M. F.; Chapeaurouge A.; Perales J.; Nirdé P.; Romanha A. J. Proteomic Analysis of *Trypanosoma cruzi* resistance to benznidazole. J. Proteome Res. 2008, 7 (6), 2357–2367. 10.1021/pr700659m. 18435557

[ref12] Machado-de-Assis G. F.; Diniz G. A.; Montoya R. A.; Dias J. C. P.; Coura J. R.; Machado-Coelho G. L. L.; Albajar-Viñas P.; Torres R. M.; Lana M. D. A serological, parasitological and clinical evaluation of untreated Chagas Disease patients and those treated with benznidazole before and thirteen years after intervention. Mem. Inst. Oswaldo Cruz. 2013, 108 (7), 873–880. 10.1590/0074-0276130122. 24037109 PMC3970640

[ref13] Gaynor P.; Gaynor D. Division of Biotechnology and GRAS Notice review Attn Re: GRAS notice for LENTEINTM Complete and Degreened LENTEINTM Complete as a nutritive ingredient in human food, 2017. https://www.fda.gov/Food/IngredientsPackagingLabeling/GRAS/NoticeInventory/default.htm. accessed 21 December 2024.

[ref14] Pradhan J.; Das B. K.; Sahu S.; Marhual N. P.; Swain A. K.; Mishra B. K.; Eknath A. E. Traditional antibacterial activity of freshwater microalga *Spirulina platensis* to aquatic pathogens. Aquac Res. 2012, 43 (9), 1287–1295. 10.1111/j.1365-2109.2011.02932.x.

[ref15] Ibañez E.; Herrero M.; Mendiola J. A.; Castro-Puyana M. Extraction and characterization of bioactive compounds with health benefits from marine resources: macro and micro algae, cyanobacteria, and invertebrates. In Marine Bioactive Compounds; Springer US: Boston, MA, 2012; pp. 55–98. 10.1007/978-1-4614-1247-2_2.

[ref16] Van Wagoner R. M.; Drummond A. K.; Wright J. L. C. Biogenetic diversity of cyanobacterial metabolites. Adv. Appl. Microbiol. 2007, 61, 89–217. 10.1016/S0065-2164(06)61004-6. 17448789

[ref17] da Silva M. R. O. B.; da Silva G. M.; da Silva A. L. F.; de Lima L. R. A.; Bezerra R. P.; Marques D. D. A. V. Bioactive compounds of Arthrospira spp. (Spirulina) with potential anticancer activities: A systematic review. ACS Chem. Biol. 2021, 16, 2057–2067. 10.1021/acschembio.1c00568. 34597512

[ref18] Abdellatief S.; Abdel Rahman A.; Abdallah F. Evaluation of immunostimulant activity of *Spirulina platensis* (*Arthrospira platensis*) and Sage (*Salvia officinalis*) in Nile tilapia (*Oreochromis niloticus*). Zagazig Vet J. 2018, 46 (1), 25–36. 10.21608/zvjz.2018.7621.

[ref19] Reboreda-Hernandez O. A.; Juarez-Serrano A. L.; Garcia-Luna I.; Rivero-Ramirez N. L.; Ortiz-Butron R.; Nogueda-Torres B.; Gonzalez-Rodriguez N. *Arthrospira maxima* paradoxical effect on *Trypanosoma cruzi*-infection. Iran J. Parasitol. 2020, 15 (2), 223 10.18502/ijpa.v15i2.3304. 32595712 PMC7311822

[ref20] Machado-Silva A.; Cerqueira P. G.; Grazielle-Silva V.; Gadelha F. R.; Peloso E. D. F.; Teixeira S. M. R.; Machado C. R. How *Trypanosoma cruzi* deals with oxidative stress: antioxidant defence and dna repair pathways. Mutat. Res./Rev. Mutat. Res. 2016, 767, 8–22. 10.1016/j.mrrev.2015.12.003. 27036062

[ref21] Coura J. R. The main sceneries of Chagas Disease transmission. The vectors, blood and oral transmissions - A comprehensive review. Mem. Inst. Oswaldo Cruz. 2015, 110 (3), 277–282. 10.1590/0074-0276140362. 25466622 PMC4489464

[ref22] Vaitkevicius-Antão V.; Moreira-Silva J.; Reino I. B. D. S. M.; de Melo M. G. N.; da Silva-Júnior J. N.; de Andrade A. F.; de Araújo P. S. R.; Bezerra R. P.; Marques D. D. A. V.; Ferreira S.; Pessoa-E-Silva R.; de Lorena V. M. B.; de Paiva-Cavalcanti M. Therapeutic potential of photosynthetic microorganisms for visceral leishmaniasis: An immunological analysis. Front. Immunol. 2022, 13, 891495 10.3389/fimmu.2022.891495. 35844611 PMC9280147

[ref23] Singh A. D.; Singh G. P. Biopigments and Rubisco expression under heavy metal stress in *Spirulina platensis*. Eco Env & Cons. 2020, 26, S351–S356.

[ref24] Nawaz M. A.; Kasote D. M.; Ullah N.; Usman K.; Alsafran M. RuBisCO: A sustainable protein ingredient for plant-based foods. Front. Sustain. Food Syst. 2024, 8, 1389309 10.3389/fsufs.2024.1389309.

[ref25] Grácio M.; Oliveira S.; Lima A.; Boavida Ferreira R. RuBisCO as a protein source for potential food applications: A Review. Food Chem. 2023, 419, 135993 10.1016/j.foodchem.2023.135993. 37030211

[ref26] Sannasimuthu A.; Ramani M.; Paray B. A.; Pasupuleti M.; Al-Sadoon M. K.; Alagumuthu T. S.; Al-Mfarij A. R.; Arshad A.; Mala K.; Arockiaraj J. *Arthrospira platensis* transglutaminase derived antioxidant peptide-packed electrospun chitosan/ poly (vinyl alcohol) nanofibrous mat accelerates wound healing, *in vitro*, via inducing mouse embryonic fibroblast proliferation. Colloids Surf., B 2020, 193, 111124 10.1016/j.colsurfb.2020.111124. 32464357

[ref27] Sannasimuthu A.; Ramani M.; Pasupuleti M.; Saraswathi N. T.; Arasu M. V.; Al-Dhabi N. A.; Arshad A.; Mala K.; Arockiaraj J. Peroxiredoxin of *Arthrospira platensis* derived short molecule yt12 influences antioxidant and anticancer activity. Cell Biol. Int. 2020, 44 (11), 2231–2242. 10.1002/cbin.11431. 32716104

[ref28] Veas R.; Rojas-Pirela M.; Castillo C.; Olea-Azar C.; Moncada M.; Ulloa P.; Rojas V.; Kemmerling U. Microalgae Extracts: Potential anti-*Trypanosoma cruzi* agents?. Biomed. Pharmacother. 2020, 127, 110178 10.1016/j.biopha.2020.110178. 32371317

[ref29] Sanchez L. M.; Lopez D.; Vesely B. A.; Togna G. D.; Gerwick W. H.; Kyle D. E.; Linington R. G. Almiramides A–C: Discovery and Development of a New Class of Leishmaniasis Lead Compounds. J. Med. Chem. 2010, 53 (10), 4187–4197. 10.1021/jm100265s. 20441198 PMC4418807

[ref30] da Silva Júnior J. N.; Oliveira K. K. D. S.; Silva A. C. D.; de Lorena V. M. B.; Marques D. D. A. V.; Bezerra R. P.; Porto A. L. F. Microalgae extracts modulates the immune response in *Trypanosoma cruzi*-infected human cells. Cytokine 2024, 179, 156621 10.1016/J.CYTO.2024.156621. 38648682

[ref31] Simmons T. L.; Engene N.; Ureña L. D.; Romero L. I.; Ortega-Barría E.; Gerwick L.; Gerwick W. H. Viridamides A and B, Lipodepsipeptides with antiprotozoal activity from the marine cyanobacterium *Oscillatoria nigro-viridis*. J. Nat. Prod. 2008, 71 (9), 1544–1550. 10.1021/np800110e. 18715036 PMC2656441

[ref32] Scarim C. B.; Chin C. M. Current Challenges and obstacles to drug development for Chagas Disease. Drug. Design. Intell. Proper. Int. J. 2018, 134 (1), 3 10.32474/DDIPIJ.2018.02.000134.

[ref33] Jantan I.; Ahmad W.; Bukhari S. N. A. Plant-derived immunomodulators: An insight on their preclinical evaluation and clinical trials. Front. Plant Sci. 2015, 6 (AUG), 158994 10.3389/fpls.2015.00655. PMC4548092 26379683

[ref34] Teixeira M. M.; Gazzinelli R. T.; Silva J. S. Chemokines, inflammation and Trypanosoma Cruzi infection. Trends Parasitol. 2002, 18 (6), 262–265. 10.1016/S1471-4922(02)02283-3. 12036740

[ref35] Vallejo A.; Monge-Maillo B.; Gutiérrez C.; Norman F. F.; López-Vélez R.; Pérez-Molina J. A. Changes in the immune response after treatment with benznidazole versus no treatment in patients with chronic indeterminate Chagas Disease. Acta Trop. 2016, 164, 117–124. 10.1016/j.actatropica.2016.09.010. 27619190

[ref36] Hunter C. A.; Ellis-Neyes L. A.; Slifer T.; Kanaly S.; Grünig G.; Fort M.; Rennick D.; Araujo F. G. IL-10 Is required to prevent immune hyperactivity during infection with *Trypanosoma cruzi*. J. Immunol. 1997, 158 (7), 3311–3316. 10.4049/jimmunol.158.7.3311. 9120288

[ref37] Álvarez J. M.; Fonseca R.; Borges da Silva H.; Marinho C. R. F.; Bortoluci K. R.; Sardinha L. R.; Epiphanio S.; D’Império Lima M. R. Chagas Disease: Still many unsolved issues. Mediators Inflamm. 2014, 2014, 912965 10.1155/2014/912965. 25104883 PMC4101227

[ref38] Cardillo F.; Voltarelli J. C.; Reed S. G.; Silva J. S. Regulation of *Trypanosoma cruzi* infection in mice by gamma interferon and Interleukin 10: Role of NK Cells. Infect. Immun. 1996, 64 (1), 128–134. 10.1128/iai.64.1.128-134.1996. 8557330 PMC173737

[ref39] Gomes J. A. S.; Molica A. M.; Keesen T. S. L.; Morato M. J. F.; de Araujo F. F.; Fares R. C. G.; Fiuza J. A.; Chaves A. T.; Pinheiro V.; Nunes M. D. C. P.; Correa-Oliveira R.; da Costa Rocha M. O. inflammatory mediators from monocytes down-regulate cellular proliferation and enhance cytokines production in patients with polar clinical forms of Chagas Disease. Hum. Immunol. 2014, 75 (1), 20–28. 10.1016/j.humimm.2013.09.009. 24071371

[ref40] Gomes J. A. S.; Bahia-Oliveira L. M. G.; Rocha M. O. C.; Martins-Filho O. A.; Gazzinelli G.; Correa-Oliveira R. Evidence that development of severe cardiomyopathy in human chagas’ disease is due to a th1-specific immune response. Infect. Immun. 2003, 71 (3), 1185–1193. 10.1128/IAI.71.3.1185-1193.2003. 12595431 PMC148818

[ref41] Piovan A.; Battaglia J.; Filippini R.; Dalla Costa V.; Facci L.; Argentini C.; Pagetta A.; Giusti P.; Zusso M. Pre- and Early post-treatment with *Arthrospira platensis* (Spirulina) extract impedes lipopolysaccharide-triggered neuroinflammation in microglia. Front. Pharmacol. 2021, 12, 724993 10.3389/fphar.2021.724993. 34566649 PMC8458903

[ref42] Garcia F. A. D. O.; Sales-Campos H.; Yuen V. G.; Machado J. R.; Viana G. S. D. B.; Oliveira C. J. F.; McNeill J. H. *Arthrospira* (Spirulina) *platensis* attenuates dextran sulfate sodium-induced colitis in mice by suppressing key pro-inflammatory cytokines. Korean. J. Gastroenterol. 2020, 76 (3), 150–158. 10.4166/kjg.2020.76.3.150. 32969363 PMC12286484

[ref43] Mullenix G. J.; Greene E. S.; Emami N. K.; Tellez-Isaias G.; Bottje W. G.; Erf G. F.; Kidd M. T.; Dridi S. *Spirulina platensis* inclusion reverses circulating pro-inflammatory (chemo)cytokine profiles in broilers fed low-protein diets. Front. Vet. Sci. 2021, 8, 640968 10.3389/fvets.2021.640968. 34041289 PMC8141556

[ref44] Mahmoud M. M. A.; El-Lamie M. M. M.; Kilany O. E.; Dessouki A. A. Spirulina (*Arthrospira platensis*) supplementation improves growth performance, feed utilization, immune response, and relieves oxidative stress in Nile tilapia (*Oreochromis Niloticus*) challenged with *Pseudomonas fluorescens*. Fish Shellfish Immunol. 2018, 72, 291–300. 10.1016/j.fsi.2017.11.006. 29117593

[ref45] Lorena V. M. B.; Lorena I. M. B.; Braz S. C. M.; Melo A. S.; Melo M. F. A. D.; Melo M. G. A. C.; Silva E. D.; Ferreira A. G. P.; Morais C. N. L.; Costa V. M. A.; Correa-Oliveira R.; Gomes Y. M. Cytokine levels in serious cardiopathy of Chagas Disease After *in vitro* stimulation with recombinant antigens from *Trypanosoma cruzi*. Scand. J. Immunol. 2010, 72 (6), 529–539. 10.1111/j.1365-3083.2010.02462.x. 21044127

[ref46] UTEX. Spirulina Medium | UTEX Culture Collection of Algae, https://utex.org/products/spirulina-medium?variant=30991737454682. accessed 15 December 2024.

[ref47] Gago A. S. Compostos bioativos de microalgas com interesse no tratamento da diabetes; 59 f Dissertation (Master in Molecular and Microbial Biology) – Faculty of Sciences and Technology, University of Algarve: Portugal, 2016. https://sapientia.ualg.pt/handle/10400.1/8557. accessed 21 December 2024.

[ref48] Laemmli U. K. Cleavage of structural proteins during the assembly of the head of bacteriophage T4. Nature 1970, 227 (5259), 680–685. 10.1038/227680a0. 5432063

[ref49] Mosmann T. Rapid colorimetric assay for cellular growth and survival: application to proliferation and cytotoxicity assays. J. Immunol. Methods 1983, 65 (1–2), 55–63. 10.1016/0022-1759(83)90303-4. 6606682

[ref50] Rashed K.; Da Silva Ferreira D.; Ferreira S.; Esperandim V. R.; Marçal M. G.; Sequeira B. M.; Gabriel L.; Flauzino B.; Cunha W. R. *In vitro* trypanocidal activity of the egyptian plant *Schinopsis lorentizii* against trypomastigote and amastigote forms of *Trypanosoma cruzi*. J. Appl. Pharm. Sci. 2016, 6 (6), 55–060. 10.7324/JAPS.2016.60610.

